# Chicago Medical Society

**Published:** 1861-03

**Authors:** 


### CHICAGO MEDICAL SOCIETY.

The regular monthly meeting of this Society, was held in their room on the 16th, March, 1861. Dr. O. Smith, President in the chair. Is the internal use of Mercurials always injurious in Scrofula? Dr. E. Andrews opened the discussion on this question, by making a distinction between the scrofulous constitution or diathesis, and the local developments connected with it. Remedies that might be beneficial or even necessary for some of the latter, might not be indicated by the former. He presumed, however, that the question referred more particularly to the use of mercury in the scrofulous constitution. He defined such constitution to consist in a peculiar delicacy of structures, with deficient tone or strength, and a special tendency to the formation of caso-plastic deposits. The preparations of Mercury are supposed to diminish the plasticity of the blood, and thereby enfeeble nutrition. Hence, they are generally regarded as injurious to those possessing a scrofulous diathesis. This, however, in his opinion did not in all cases forbid the use of mercurials in the treatment of local inflammations, occurring in such subjects. They might be necessary, and promptly beneficial, in the treatment of inflammation of the internal structures of the eye, even in scrofulous constitutions; but in such cases he would deem it necessary to be more cautious, about the long continued or excessive use of this class ef remedies.

Dr. E. L. Holmes, regarded the use of Mercurials, in a cautious and proper manner, not only as admissable, but as beneficial, in many cases of scrofulous Ophthalmia of children. He had often used, in such cases, the Hydrarg. Cum Creta, and small doses of the Bichloride Hydrarg. in connection with tonics, and without any immediate or remote effects of an injurious character.

Dr. S. Wickersham, inquired whether the use of mercurials in the treatment of scrofulous inflammations in children, did not increase the danger of the formation of tubercular deposits at a subsequent period. He thought he had seen cases, in which scrofulous enlargements of the external lymphatic glands had disappeared under the use of mercurials, and the same patients were not long afterwards manifesting signs of pulmonary tuberculosis. He entertained doubts whether mercurials could be used in any case of scrofula, without injurious effects, either present or future.

The discussion was furthercontinued, by Drs. C. G. Smith, N. S. Davis, and O. Smith.

Dr. A. Fisher, read an interesting paper on strangulated hernia. Two cases were detailed, one of which was of peculiar interest; but as the paper will be published entire in our next number, we will not attempt an analysis of it here.

